# Successful treatment of recalcitrant pityriasis lichenoides et varioliformis acuta with baricitinib: A case report

**DOI:** 10.1016/j.jdcr.2026.05.035

**Published:** 2026-05-22

**Authors:** Nouf AlMuhanna, AlAnoud A. AlSalman, Mishari Tariq Alrubaiaan, Mohammed Abdulaziz AlOsaimi, Nouf Abdulaziz Almagushi, Ahmed Alhumidi

**Affiliations:** a Department of Dermatology, King Fahad Medical City, Riyadh, Saudi Arabia; b College of Medicine, King Saud bin Abdulaziz University for Health Sciences, Riyadh, Saudi Arabia; c Department of Pathology, King Saud University, Riyadh, Saudi Arabia

**Keywords:** baricitinib, Janus kinase inhibitor, pityriasis lichenoides, pityriasis lichenoides et varioliformis acuta, PLEVA

## Introduction

Pityriasis lichenoides et varioliformis acuta (PLEVA) is a rare inflammatory papulosquamous condition with recurrent erythematous papules that develop in crops and may subsequently deteriorate into vesiculating, necrotic, or crusted lesions that evolve almost always to varioliform scars with a relapsing clinical course. Although the cause is unclear, there has been growing evidence of cytotoxic T-cell-mediated immunological dysregulation in PLEVA. Recent studies have shown T-cell receptor clonality in some cases, substantiating the existence of T-cell populations within the lesions. Raghavan et al reported 12 cases and 17 histopathological specimens with pityriasis lichenoides (both PLEVA and Pityriasis lichenoides chronica [PLC].^1^ T-cell clonality was identified in 58% of cases and 53% of histopathological specimens. In addition, colonality was observed more frequently in PLEVA than in PLC.^1^ Histologically, PLEVA shows interface dermatitis with lymphocytic exocytosis and necrotic keratinocytes, along with a band-like dermal lymphocytic infiltrate compatible with an immunologic response.^2^^,^^3^ Treatments vary between macrolides, tetracyclines, narrowband ultraviolet B (UVB) treatment, and systemic corticosteroids. More severe disease may require immunosuppression with methotrexate or cyclosporine; however, the response is only partial, and often transient.^4^^,^^5^ An unusual cytokine pathway with Janus kinase/signal transducer and activator of transcription (JAK/STAT) signaling has emerged as a significant contributor to multiple T-cell-mediated dermatoses over the past several years.^6^ Baricitinib is an oral selective JAK1/JAK2 inhibitor that has shown efficacy in treating atopic dermatitis, alopecia areata, and psoriasis, and is being used off-label with increasing frequency in other inflammatory dermatoses.^7^ At the time of publication, its use for PLEVA has not been widely reported, to our knowledge. Thus, we report a case of recalcitrant PLEVA that improved with baricitinib, suggesting a potential role for JAK inhibition as an alternative treatment for this intractable disorder.

## Case report

**Fig 1:**
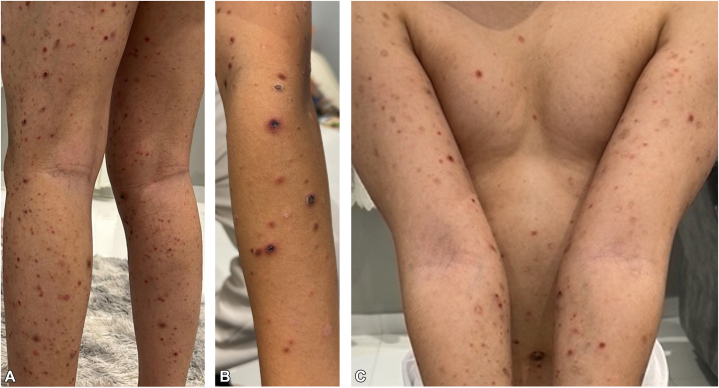
There are numerous pink-red papules with overlying fine scales, superficial erosions covered with hemorrhagic crust on the **(A)** bilateral lower extremities, **(B)** upper extremities, **(C)** chest.

**Fig 2:**
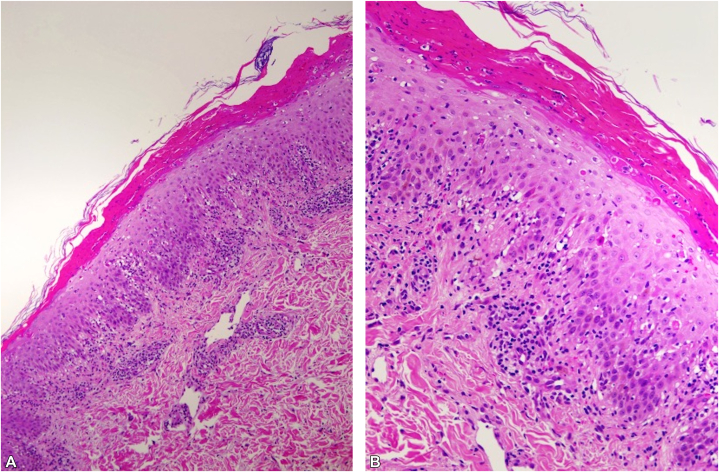
Microscopic examination of the skin punch biopsy at **(A)** ×200 and **(B)** ×400 magnification, showing focal parakeratosis and acanthosis with scattered necrotic keratinocytes, a superficial perivascular and interface lymphocytic infiltrate is present, with extravasated RBCs, and exocytosis of small lymphocytes into the epidermis after staining with hematoxylin and eosin.

**Fig 3:**
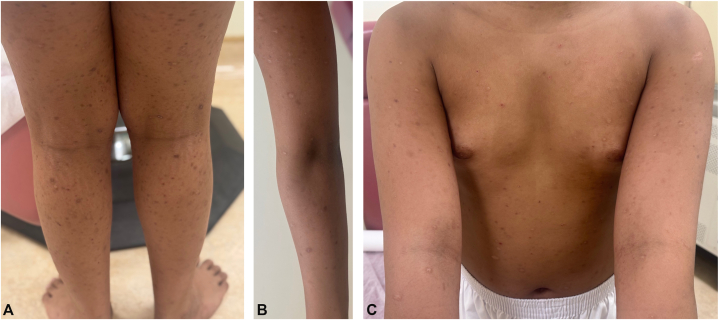
This figure shows complete clearance of the lesions over bilateral **(A)** lower and **(B)** upper extremities, **(C)** chest after treatment with baricitinib 2 mg daily.

A 12-year-old boy with a history of recurrent tonsillitis requiring multiple courses of antibiotics presented with a generalized pruritic skin eruption lasting 2 years. The eruption began 2 weeks after the tonsillitis. Initially, lesions appeared on the upper extremities and then spread across the body, associated with severe itching that affected his daily activities. There was no history of atopy, or drug or food allergy. Examination revealed numerous pink-red papules with overlying fine scales, and superficial erosions covered with hemorrhagic crust on the chest, back, and bilateral upper and lower extremities (Fig 1). Laboratory investigation results were unremarkable. A skin punch biopsy taken revealed focal parakeratosis and acanthosis with scattered necrotic keratinocytes. A superficial perivascular and interface lymphocytic infiltrate is present, with extravasated RBCs, and exocytosis of small lymphocytes into the epidermis after staining with hematoxylin and eosin; thus, PLEVA was confirmed (Fig 2). The patient received multiple treatments without improvement, and the condition worsened. These treatments included clarithromycin 250 mg once daily for 1 month, methotrexate 10 mg once weekly, folic acid with mometasone furoate ointment for 3 months, and tofacitinib 5 mg twice daily for 3 months, along with clobetasol cream. Then the patient was started with narrowband UVB phototherapy twice weekly, doxycycline 100 mg daily for 3 months, betamethasone valerate ointment twice daily for 2 weeks, and topical tacrolimus for 2 weeks but these led to no improvement. After baseline labs, baricitinib 2 mg daily was started for 1 month. The response to this treatment was satisfactory, with remission of the lesions and a significant reduction in pruritus (Fig 3).

## Discussion

We present a 12-year-old boy with recalcitrant PLEVA who failed several systemic and topical therapies. After starting the JAK inhibitor, baricitinib, clinical improvement was substantial with no active papules on follow-up, underscoring the potential role of JAK inhibitors in such instances. Standard treatment strategies for PLEVA include phototherapy, antibiotics, and methotrexate. A systematic review by Bellinato et al found narrowband UVB is an optimal first-line treatment.^8^ Yet, recurrence after treatment discontinuation and ongoing remission were difficult to accomplish. Conversely, macrolides and tetracyclines, functioning through anti-inflammatory pathways rather than their antimicrobial properties, may offer some benefits; however, response rates remain inconsistent and incomplete. A viable alternative for severe and recurrent cases is methotrexate. However, it is limited by the necessity for frequent systemic monitoring and the potential risk of immunosuppression.^8^ In general, to date, no single therapy has sufficient evidence, and most recommendations are based on retrospective investigations. The above treatment strategies correspond with the case of our patient, who received macrolide antibiotics, topical corticosteroids, and narrowband UVB, resulting in no improvement and worsening of the disease course, illustrating the necessity for an alternate therapeutic strategy. Due to the recent recognition of the significance of T-cell activation and cytokine signaling in PLEVA, JAK Inhibitors provided an appropriate alternative in our instance.^1^ Recent reports demonstrated that JAK 1/2 inhibitors improved pityriasis lichenoides, specifically upadacitinib and ruxolitinib, when traditional treatments failed. Houpe et al reported that upadacitinib successfully cured bullous PLEVA at a dosage of 15 mg daily, following the administration of deucravacitinib and dupilumab which led to worsening of PLEVA.^9^ Likewise, Wu et al documented a case of Febrile Ulceronecrotic Mucha-Habermann Disease, a rare and severe type of PLEVA, which rapidly resolved following the administration of ruxolitinib and methotrexate, raising doubts concerning individual versus combinational therapy efficacy.^10^ In our case, following the initiation of baricitinib, a significant improvement in the count and activity of lesions was noted, resulting in complete clinical resolution. However, to date, reports documenting the successful application of JAK inhibitors remain exceedingly rare. To our knowledge, this is the first report documenting the effective use of baricitinib in a patient with PLEVA, highlighting the emerging value of JAK Inhibitors as targeted immunomodulators in pityriasis lichenoides disorders. Yet, to fully assess JAK inhibitor efficacy and safety in pityriasis lichenoides diseases, large multicentric trials with longer follow-up are needed.

## Conflicts of interest

None disclosed.
